# Communicating with children and adolescents about the risk of natural disasters

**DOI:** 10.1080/20008198.2018.1429771

**Published:** 2018-02-06

**Authors:** Liv Gunvor Hove Midtbust, Atle Dyregrov, Heidi Wittrup Djup

**Affiliations:** ^a^ Center for Crisis Psychology, Faculty of Psychology, University of Bergen, Bergen, Norway

**Keywords:** Natural disasters, risk communication, resilience, children, adolescents, desastres naturales, comunicación de riesgos, resiliencia, niños, adolescentes, 自然灾害, 风险交流, 韧性, 儿童, 青少年, • This article presents an overview of relevant literature regarding how to communicate with children and adolescents about natural disasters.• Risk communication and disaster education can be offered at schools, through parental guidance, as informational campaigns and by using Internet and social media. More needs to be known about the influence of social factors on children’s risk perception and responses.• Children can be protagonists for action to reduce disaster risk in their communities. They can inform programmes concerning risk, as well as providing guidance about their needs following a disaster.• Suggestions for further investigations are offered, especially to clarify whether the various strategies promote behavioural changes.

## Abstract

A vast number of people annually are affected by natural disasters. Children are at risk of losing their lives and suffer mentally or physically after such events. The fostering of resilience and preparedness ahead of disasters can reduce untoward effects of disastrous events. Risk communication and disaster education are considered important aspects of disaster preparedness, but little is known about whether such strategies influence children’s behaviour when natural disasters occur or how they cope in the aftermath. This paper presents and discusses various strategies that promote preparedness activities to save lives. To a minor extent, it also includes strategies that can promote coping in the aftermath. Strategies such as informational campaigns, educational activities, psychoeducation and parental guidance are addressed. The literature to date indicates that schools are a suitable arena for risk communication, and that adolescents themselves should be involved and engaged in the communication strategies. However, the relationship between knowledge of preparedness *strategies* and the resulting preparedness *actions* is largely unknown. It is unknown whether changes in awareness and attitudes have resulted in actual behaviour change. It is advocated that preparedness activities and parental involvement should supplement information-based strategies.

## Comunicarse con niños y adolescentes sobre el riesgo de desastres naturales

Un gran número de personas se ven afectadas anualmente por desastres naturales. Los niños corren el riesgo de perder la vida o sufrir mental o físicamente después de dichos eventos. Promover la resiliencia y la preparación ante los desastres puede reducir los efectos desfavorables de los sucesos desastrosos. La comunicación de riesgos y la educación para desastres se consideran aspectos importantes de la preparación para casos de desastre, pero se sabe poco acerca de si tales estrategias realmente influyen en el comportamiento de los niños cuando ocurren desastres naturales o cómo lidian con ello después. Este documento presenta y discute varias estrategias que promueven actividades de preparación para salvar vidas. En menor medida, también incluye estrategias que pueden promover el afrontamiento posterior. Se abordan estrategias como campañas informativas, actividades educativas, psicoeducación y orientación parental. La literatura hasta la fecha indica que las escuelas son un entorno adecuado para la comunicación de riesgos, y que los propios adolescentes deberían estar involucrados y comprometido en las estrategias de comunicación. Sin embargo, la relación entre el conocimiento de *las estrategias* de preparación y *las acciones* de preparación resultantes es en gran parte desconocida. Se desconoce si los cambios de conciencia y actitudes han resultado en un verdadero cambio de comportamiento. Se recomienda que las actividades de preparación y la participación de los padres complementen las estrategias basadas en la información.

## Abstract (zh)

每年有大量受害者被自然灾害影响。这给儿童带来丧生风险和身心问题。在灾害发生前就培育韧性和做好准备工作，可以减少灾难事件的不幸影响。风险沟通和灾害教育是灾害准备中的重要一环，但还不清楚这些策略是否可以在自然灾害实际发生时实际影响儿童的行为，以及事后儿童的应对。本文呈现并讨论可以增进准备行为的多种策略，以挽救更多生命。也包括了一小部分在灾害后可以促进应对的一些策略。文中还提到了如知识性宣讲、教育性活动、心理教育和父母指导一类的策略。前人文献指出学校是风险交流的合适场所，而且青少年本身也应该参与到交流的策略中来。但是还不清楚关于准备工作的知识和实际准备行为之间的相关性，以及意识和态度上的改变是否会转变成最终的行为改变。在基于信息的方式以外，还提倡更多准备行为和父母参与。

## 1. Introduction

There has been an exponential increase in the number of natural disasters and people affected by them (Codreanu, Celenza, & Jacobs, 2014). It is estimated that 175 million children per year will be affected by natural disasters attributed to climate change (Codreanu et al., 2014). An unknown number of children die each year, and many children experience physical injuries and mental health effects following natural disasters. It is estimated that 5–43% of affected children will experience posttraumatic stress disorder (PTSD), and many will suffer from depression, anxiety and other mental health effects (Kar, 2009). The number of affected children deems it important to develop steps to increase survival and mitigate aftereffects. Considering the vast number of children at risk of suffering severe effects due to natural disasters, steps should be taken to foster survival and resilience among this group.

Communication is an important part of disaster and emergency management and response. Houston (2012) distinguishes between risk communication and crisis communication. While *risk communication* focuses on influencing individual understanding of and behaviour related to risk, *crisis communication* deals with how to respond and cope with a particular event.

The major focus of this paper will be on how risk communication can act to secure survival during a disaster. It will also briefly discuss how information, either before or in the aftermath of a disaster, can lessen the mental effects of natural disasters on children and adolescents.

This paper is a narrative review article (Uman, 2011). Though systematic searches in PsycINFO have been conducted, together with more simple searches in Google Scholar, we have deliberately made a choice of studies that we believe illuminate this new research area.

## 2. Disaster risk communication: interventions to foster actions, coping and resilience

Disaster communication interventions are argued to be effective, yet often overlooked, tools in achieving mental and behavioural outcomes (Houston, 2012). Although these efforts often are included in risk communication models, Houston (2012) called for a comprehensive list of disaster intervention activities and an incorporation of this into a multiphase framework. Based on a literature review, he proposed a Disaster Communication Intervention Framework (DCIF) to include many public health outcome targets and strategies on how to reach them. He suggested that this framework could give direction to future research, for example in developing and evaluating packaged and standardized disaster communication interventions. The aim for the DCIF is ‘improving individual and community preparedness and resilience; decreasing disaster-related distress; promoting wellness, coping, recovery and resilience; helping a community make sense of what happened during and after a disaster; and rebuilding the community’ (Houston, 2012, p. 283). According to Houston, a variety of strategies can be used to achieve these outcomes, and which strategy to choose depends on which phase of a disaster one finds oneself in. Houston (2012) does not distinguish clearly between teaching children survival strategies and strategies that promote coping after the event. While the first group of strategies could be seen as actions to be taken to survive, such as getting under a desk or table during an earthquake, the use of inner mental strategies to cope with aftereffects are quite different. The challenges may be much more extensive in teaching children the latter type of strategies. Although the lines between the different phases of a disaster – before, during and after – are not always clear-cut (Houston, 2012), we will focus primarily on pre-event communications and programmes.

### 2.1. Communication before a disaster

Houston (2012) argues that in the pre-event phase, the most important aim in risk communication is to improve the levels of individual and community ‘preparedness’ and ‘resilience’. Preparedness is defined as ‘activities undertaken before a disaster that are intended to help individuals, families, and communities respond to and cope with a disaster when an event occurs’. Resilience, on the other hand, is ‘a process linking a set of adaptive capacities to a positive trajectory of functioning and adaptation after a disturbance’ (Houston, 2012, p. 286). To increase these capacities, Houston (2012) suggests various strategies. Offering the public education about risk, and promoting activities that increase preparedness and reduce risk, are key elements of disaster communication in the pre-event phase. The information must be credible, current and helpful. For example, it is recommended to inform the public about existing disaster plans at local and/or governmental level. The strategies should also aim to increase a community’s collective efficacy and resilience by developing and strengthening community connections and relationships, and to engage the community in discussions about risk, disaster plans and response.

A suggested pre-event strategy is to prevent post-event distress by providing ‘psychological vaccines’ to individuals or a community. This involves offering psychoeducation to children and adults about normal reactions to disasters, so that their responses to disasters are more informed and adaptive when they occur (Houston, 2012). Psychoeducation normally focuses on common psychological reactions and stress management techniques (Pfefferbaum & Shaw, 2013). For parents, however, it is important to also provide them with information about children’s typical reactions and symptoms that could indicate a need for further evaluation.

It is unclear if Houston suggests that all children in schools should receive information about normal reactions to disasters or if this is to be conducted in high risk areas. The amount of potentially traumatic events that children are exposed to during childhood is very high, with a US lifetime prevalence of 62% (McLaughlin et al., 2013). A general approach where all children are provided with information about usual reactions to potentially traumatic or disastrous events could be argued for. However, alerting them to possible reactions may cause undue fear and even the risk of self-fulfilling prophesies.

### 2.2. Disaster education programmes

Schools constitute a suitable arena for disaster communication interventions directed towards youth. At a governmental level, this has led to disaster and risk reduction education and activities at schools (Codreanu et al., 2014). These are geared towards increasing survival. In some countries (e.g. the US), the importance of risk education is recognized at a governmental level, resulting in a greater awareness of children’s needs. In others (e.g. Indonesia), disaster education in public schools has not been introduced even after devastating events like the 2004 tsunami (Codreanu et al., 2014). Although many schools in the US are mandated to hold drills to assure rapid, correct responses following earthquakes, it has not been assessed whether they are effective in improving preparedness (Ramirez, Kubicek, Peek-Asa, & Wong, 2009). There have been some attempts to evaluate education exercises in preparation for earthquakes (Johnston et al., 2011), but it is yet not known if this transfers to optimal behaviour during a real event. Whether interventions attempting to increase preparedness leads to actual behavioural changes is not only relevant when it comes to increasing the chance of survival; it may also have a positive outcome on mental health. It is believed and shown in the research literature on self-efficacy that being able to act on one’s environment is related to good coping (Bandura, 1997). From our clinical experience in meeting survivors of disastrous events, we have observed how taking actions to survive has had positive effects on mental health. Consider the following example from our own clinical experience:

Two brothers 12 and 10 years of age survived a fire. While the 12-year-old told his brother that they should hide under the bed-cover, the 10-year-old took action and had them escape out to the roof to be saved by the fire service. Following the event, the younger brother was almost bursting with pride in his achievement, while the older brother seriously doubted himself and his ability to cope in a crisis (Clinical case from second author).

There has not been much research in this area, but it is known that giving children responsibility to care for others and encouraging active coping can make them less vulnerable during stressful periods (Clarke, 2006). Furthermore, young children who took on a caregiver role towards a huggy-puppy doll during a war situation had their stress reaction alleviated (Sadeh, Hen-Gal, & Tikotzky, 2008). The link between actions and post-event mental effects in children furher explored through research.

Some studies, however, do suggest that experiential, community-based activities are more effective in promoting health-related activities and civil engagement than information-based education to initiate preparedness activities such as risk appraisal, decision for prevention action and risk-reducing behaviour (Jang, Johnson, & Kim, 2011; Shiwaku, Shaw, Kandel, Shrestha, & Dixit, 2007). That is, although lectures may increase risk perception, they do not automatically enable youth to know the importance of pre-event preparations and to take action to reduce disaster risk. It is therefore argued that school education programmes should be both active and involve the community (Shiwaku et al., 2007). Furthermore, an individual’s resilience is not only a matter of theoretical knowledge, but also calls for many forms of learning (visual, verbal, logical, mathematical), organizational skills, spatial thinking and accurate decision making (Codreanu et al., 2014). Educational disaster interventions may therefore be more effective if they include other ways of learning than the traditional teacher-to-student, passive knowledge-acquisition format.

One way to be more active is to encourage the children/adolescents to discuss a mock disaster. This can be done through a tabletop exercise or a classroom discussion. Talking through what they should do in the event of a disaster can be followed by completing the necessary actions to be taken, i.e. evacuate the classroom or enter safe zone.

Houston (2012), as part of his DCIF strategy, also advocates to inoculate children against post-event distress by providing them with a ‘psychological vaccine’. He adheres to the view of Friedman (2005), that this vaccine can work by giving children information about expected reactions to make their response more informed and adaptive. By tabletop exercise or classroom discussion, children can be given an opportunity to emotionally and cognitively process potentially traumatizing information ahead of an event (Houston, 2012). It is argued that ‘people who have gone through a fearful “adjustment reaction” before a crisis begins are prepared to cope with the crisis, emotionally as well as logistically’ (Sandman, 2006, p. 259). It should be noted, however, that, to our knowledge, there have been no experimental studies of the effect of such psychological vaccines and it is therefore unknown whether this will have a positive effect on coping after a disaster. It may even increase fear of such events taking place. The results from research on ‘vaccinating’ adults serving on military or humanitarian missions or participating in disaster work have been mixed, although mostly positive (Brooks, Dunn, Amlôt, Greenberg, & Rubin, 2016; Mulligan, Fear, Wessely, & Greenberg, 2011). Whether this will hold for children and adolescents is not known.

Fear appeals can produce at least two different outcomes: (1) an emotional, defensive ‘fear control’ response, where the receiver denies or minimizes the risk or responds with even more dysfunctional, risk-seeking behaviour; or (2) a cognitive, preventive ‘danger control’ response, where the recipient acts to mitigate the risk (Ryan, Hocke, & Hilyard, 2012; Witte, 1992). If the child feels capable of managing the threat, i.e. the message includes something about what can be done, or the child has pre-disaster knowledge about what to do, appropriate behaviour can ensue. However, scaring children who do not have the tools to act will only result in unhealthy fear (Witte, 1992). Fear appeal directed towards children should be balanced with healthy parent–child discourse to foster coping and learning (Ryan et al., 2012).

Fraustino and Ma (2015) examined a humorous disaster-preparedness message that spread through social media, involving how to prepare for a zombie apocalypse. This was a viral hit and reached many people in a very brief time. However, the results showed that the humorous appeal resulted in a weaker intention to take preparedness actions than did non-humorous message strategies. Moreover, the humorous strategies caused significantly weaker intentions to seek additional emergency information in comparison to the non-humorous strategies (Fraustino & Ma, 2015). Using a humorous appeal might draw attention to and increase the liking of a campaign, but runs the risk that people do not take the message seriously. Most of the studies in this area have looked at ‘students’, not children and teenagers. Consequently, we cannot be sure that the use of humour would produce the same results for children and adolescents.

An additional approach involves the use of video games. Children and young adults are extensive users of digital devices, and video games could reach a large and diverse audience. To our knowledge, there have been no attempts to communicate disaster risk by using this strategy, but several studies have demonstrated positive health-related changes from playing video games addressing health promotion in areas such as diet, physical activity and asthma (Baranowski, Buday, Thompson, & Baranowski, 2008). To be successful, the games need to be appealing, effective and affordable.

### 2.3. Evaluation and effects

#### 2.3.1 Does knowledge translate into preparedness actions?

Codreanu et al. (2014) argues that, after about two decades of increased attention towards the development of disaster resilience, it is necessary to ask whether the current methods of disaster education of the teenage population (1) enhances their knowledge of skills in disasters, and (2) translates into behavioural changes improving their chances for survival. Contemporary research in risk communication has found that the relationship between knowledge of preparedness *strategies* and preparedness *actions* is not necessarily very strong. Education programmes might contribute to a change in awareness and attitudes towards risk, but this does not necessarily mean that people’s behaviour actually changes (Jacobs, Sisco, Hill, Malter, & Figueredo, 2012; Johnson, Ronan, Johnston, & Peace, 2014). Furthermore, the effects evaluated are only short-term effects, and little is known about the long-term effects of disaster education (Ronan, Alisic, Towers, Johnson, & Johnston, 2015).

To be able to say more about the effects of such programmes, it is necessary to have more studies that include outcome measures on behavioural change. This is of course a challenge, given that it is not easy to predict when and where there will be a natural disaster. Thus, it is difficult to gather pre-disaster measures, and the chaos following a disaster precludes data-collection post-disaster.

#### 2.3.2. Does knowledge increase anxiety and/or resilience?

According to a review of Johnson et al. (2014), several studies have aimed to measure the emotional impacts of disaster education programmes on children. The results were somewhat mixed. Overall, most of them concluded that the programmes had no significant impact on children’s reported levels of fear. However, an evaluation of Save the Children’s Ready and Resilient programme found that about half of the participants reported increased worry about the disasters after the programme. The authors did however conclude that this result could not be interpreted as either a positive or a negative outcome, since anxiety has been associated with a higher coping potential and household preparedness (Blanchet-Cohen & Nelems, as cited in Johnson et al., 2014).

Ronan, Johnston, Daly, and Fairley (2001) found that children who demonstrated unrealistic risk perceptions (e.g. believing that low-frequency risks have a high rate) had more hazard-related fears and showed lower levels of confidence in their ability to cope emotionally with a future hazard, compared to children with more realistic risk perceptions. Moreover, the fearful group of youths had lower levels of knowledge about emergency response compared to less fearful children (Ronan et al., 2001). The differences in knowledge and fear were explained by whether they had been exposed to school-based hazard education programmes or not. That is, the children who had received education were more knowledgeable and less anxious (Ronan et al., 2001).

Ronan and Johnston (2003) conducted a quasi-experimental study investigating the effects of hazard education programmes for youth on anxiety, coping strategies and resilience. They had two conditions in their study. The usual condition (UC) involved a six-week long module of structured reading and discussion programmes about the subject ‘Disaster’. The emergency management condition (EM) included the same material as the UC condition, but also explicit guidance about hazard mitigation and emergency response. In addition, it included interaction between youths and parents aimed at increasing hazard adjustment activities at home. The researchers found that the EM condition outperformed the UC condition when considering effects on problem-focused coping (i.e. hazard-adjustment, readiness, emergency management knowledge), but not on emotion-focused coping (i.e. hazard-related fears, perceived parental distress, perceived emotional coping). Both groups displayed reduced levels of fear and perceived parental distress.

Ronan and Johnston (2003) support an emphasis on emergency management in hazard education and argue that the research to date supports that teachers and parents should discuss hazards and disasters with children to increase resilience. This might be particularly important for disasters that receive wide media coverage. It is argued that when something is widely published, it is unfortunate if adults avoid discussing these issues. This is supported by studies that show that children tend to see more television news than parents believe (Smith & Wilson, 2000), and that news on TV can produce distress in children (Korhonen & Lahikainen, 2008; Smith & Wilson, 2000). By avoiding these topics, youth might experience increased anxiety about the events and they may also develop potentially inaccurate perceptions of adult’s feelings about the events. Considering that children often rely on adults for coping with problems, it is important that adults are willing to discuss such events and, in doing so, provide a ‘coping model’ for children and adolescents.

Although the results are promising concerning increased resilience in children following educational programmes, there still is a dearth of data-based literature in the area. While there are some studies supporting the use of brief programmes in the aftermath of a disaster, little research has assessed factors related to children’s risk perceptions and preparedness and the role of hazard education programmes prior to a disaster (Ronan & Johnston, 2003). Although children do not have the same level of independence of action as adults do, it is important to include information that helps a child understand what he or she can do physically and emotionally to prepare for a disaster, and what they would need help from others to manage.

## 3. Factors that contribute to how children and adolescents perceive risk and respond to risk communication

### 3.1. Individual factors

There are several factors that might contribute to how youth perceive and are affected by risk communication and disaster education programmes. It is not clear if and how age, gender and ethnicity influence preparedness and risk perceptions (Johnson et al., 2014). Some studies suggest that there is a link between prior disaster experience and risk perceptions (Halpern-Felsher et al., 2001; Johnson et al., 2014), whereas other studies do not find a significant effect of disaster experience on respondents’ disaster knowledge (Johnson et al., 2014).

We also lack research that elucidates how a person’s cognitive and self-regulating skills and other individual characteristics may influence their perception of risk and survival during an actual disaster. However, we have more knowledge about factors associated with coping and resilience in the aftermath of a disaster (Codreanu et al., 2014). Individual factors such as age, education, previous level of trauma exposure, life stressors, health and social and familial support are associated with better coping (Pfefferbaum, Jacobs, Houston, & Griffin, 2015; Pfefferbaum et al., 2016). Unfortunately, the concept of coping and inconsistencies in coping dimensions across studies makes it difficult to know how individual coping affects long-term reactions.

### 3.2. Family and network factors

How parents think and respond to disaster risk influences children and adolescents. Previous research has shown that survival likelihood is higher in parents with prior knowledge of earthquakes, and that this directly affects their children’s understanding and knowledge (Codreanu et al., 2014). Children may follow their parents’ advice to survive, and also model the behaviour of their parents. Knowledgeable parents may therefore secure increased survival for both themselves and their offspring.

Regarding psychological aftereffects of the disaster, how a parent reacts to the disaster may directly affect the child’s coping. Children who perceive greater levels of distress in their parents seem to cope less effectively in the aftermath of a disaster. Parental distress is thought to be a moderator of children’s own fear. Interventions aiming to decrease distress among parents are therefore likely to be beneficial for youth (Ronan & Johnston, 2003).

Supportive and stable families improve young people’s responses to stress, and factors that contribute to resilience include parental qualities such as warmth, responsiveness, stimulation, spending time with the children and consistent guidance and rules (Hill, Stafford, Seaman, Ross, & Daniel, 2007). Even though parenting style might be a protective factor for an individual’s resilience (Codreanu et al., 2014), this depends on whether the parents have an inclusive communication style where they engage their children in disaster discussions and disaster preparation. Several studies support the link between parental distress and parenting style (Bauer, Burch, Van Abbema, & Ackil, 2007; Sales & Fivush, 2003), which is important to be aware of when planning disaster interventions. It is beneficial to encourage parents to talk to their children about a disaster. If they struggle to do so, it might be best that other adults provide the children with the information they need to make sense of what has happened. It is recommended that parents should not avoid difficult topics or uncomfortable conversations, but rather listen to their children, reassure them and correct any misunderstandings they might have (Child Welfare Information Gateway, 2014). Children’s engagement with parents both facilitates knowledge transfer from children to parent, and improves the quality of children’s learning (Codreanu et al., 2014; Johnson et al., 2014). Moreover, research has found that increased interaction between children and parents in hazards education programmes predicts readiness at home, measured in increased number of hazard adjustments (Ronan & Johnston, 2003).

By researching how parental risk perception affects children’s perceptions, we may increase our knowledge and better understand how to communicate risk to children.

### 3.3. Local society and public society factors

Children and adolescents are residents of both smaller and larger societies, and how these societies perceive and think about disasters affect everyone living in them. Disaster awareness, disaster-mitigation planning and proactive participation might be influenced by the disaster history of populations in risk prone areas (Codreanu et al., 2014; Rød, Botan, & Holen, 2011). For example, in areas where landslides have taken place previously, the events may continue to live on in people’s collective memory and become a part of children’s upbringing, influencing their perception of risk.

How the community responds to a disaster or disaster risk also affects how people living in that area cope with the situation and hazards. Codreanu et al. (2014) state that the characteristics of a competent community in promoting resilience are critical reflection, flexibility, creativity in problem solving, decision-making, conflict negotiation, resource acquisition and protection, advocacy and collaboration in community action. In contrast, disadvantaged communities are disintegrated, disenfranchised and lack expertise (Codreanu et al., 2014). We assume that in these communities this adds to the negative aftereffects in both children and adults.

Like the resilience of a person, the resilience of a community is not a fixed capacity and is therefore possible to increase through the right actions. Thus, several interventions directed towards increasing community resilience have been proposed during the last decades (Pfefferbaum, Pfefferbaum, & Horn, 2015).

## 4. Disaster risk communication and pre-event strategies: implementations and recommendations

The literature is scarce when it comes to disaster risk communication directed towards children and adolescents. We here put forward some reflections and recommendations on how to communicate with children and their parents about disaster risk and mental health consequences.

### 4.1. Reaching children and adolescents

Schools are a suitable arena for disaster communication strategies both when it comes to providing youth with valuable information about disaster risk and to train them in active coping strategies before a disaster occurs. In addition to (or as an alternative to) schools and kindergarten, psychoeducation should be provided in places where children and families naturally congregate, such as sport activities, faith-based gatherings, community meetings and primary care settings. Disaster education can involve school education, self-education, community education and family education. Behavioural change in disaster preparedness has been observed to result mostly from community and family education (Codreanu et al., 2014). We suspect that the most effective way of communicating with children will be using several platforms or modes of communication.

Research has indicated that, in risk communication, a one-way communication from an authority towards a particular group may not be the most beneficial. Campaigns stimulating interpersonal and two-way communication about the campaign topic are more effective when it comes to learning, and results in more individual attitude and behaviour changes compared to a one-way campaign message alone (Houston, First, Spialek, Sorenson, & Koch, 2016). Normally, however, campaigns that target large audiences must rely on communication through channels such as the Internet, radio and television to reach their audience. By trying to connect individuals, and foster dynamic social relationships, campaigns may become more effective.

### 4.2. Social influence on perceptions of risk

People’s risk perceptions seem to be affected by social influence (Knoll, Magis-Weinberg, Speekenbrink, & Blakemore, 2015). People decide on their reaction from listening and watching what others say and do. Though younger children are heavily influenced by their parents or caretakers, adolescents increasingly look to their friends and peers to interpret and respond to what happens around them.

When being told how other people rate a risk, people tended to change their ratings in the direction of other people’ ratings. All age groups, except 12–14-year-olds, are more influenced by adults, which is consistent with other studies showing that expertise and status are strong predictors of social influence (Driskell & Mullen, 1990; Engelmann, Moore, Monica Capra, & Berns, 2012; Jetten, Hornsey, & Adarves-Yorno, 2006). When it comes to the young adolescents, on the other hand, this group values more what other teenagers say (Knoll et al., 2015). It is likely, therefore, that interventions directed towards younger age groups should emphasize social influence to a larger extent and adjust the programmes accordingly. To our knowledge, there is a lack of knowledge about how youth discuss and talk about disaster risk with each other. This is an area open for investigation.

There is growing evidence for the ability of children to act as protagonists for action to reduce disaster risk in their communities (Institute of Development Studies, 2009). Children have a unique perception of risk and they can communicate these perceptions of risk to others. In this way, they may be able to bring about changes that will reduce risk vulnerabilities (Institute of Development Studies, 2009). This challenges the traditional view in risk communication, where experts inform the public and adults are assumed to be attuned to their families’ needs and able to act accordingly and appropriately. Increasingly, children are heard regarding their needs following disasters. Following the 2004 Tsunami in South East Asia, Plan consulted children to better understand their long-term needs. They concluded that the active engagement of children mitigated the impact of loss of loved ones and assets resulting from natural disasters, and that their involvement was essential to the recovery of the community in the short, medium and long term (Plan, 2005). There is no reason to think that children’s voices cannot be heard more clearly about pre-disaster communications.

Children can be engaged in co-constructing the knowledge needed to communicate risk by placing information within their own reality. This empowers children and echoes research showing that communication and interaction with other members of the community can be crucial in creating the active support and behavioural changes that reduce disaster risks (Institute of Development Studies, 2009). Well-informed youth can network within their community, they are trusted by their peers and can function as politically neutral actors that dispel competing beliefs and convince adults about new risks (Mitchell, Tanner, & Haynes, 2009).

### 4.3. Mitigation of effects after the disaster

Disasters can have both short- and long-term consequences for the psychological health of children. In a review of studies by Kar (2009), the prevalence of PTSD was 5–43%. Comorbidities were common, especially with depression and anxiety. Maclean, Popovici, and French (2016) found a clear association between having experienced a natural disaster before the age of five and mental health difficulties, especially anxiety, in adult life. Most interventions following disasters use a public health approach to reach many. They often start with less intensive interventions, such as information to all affected individuals, and then use a stepped care model, where reactions are monitored and help is aligned to their needs and requirements (McDermott & Cobham, 2014).

Outreach efforts include communicating information about common, normal reactions (psychoeducation) to children and parents. Information can be communicated directly to children in schools or in community meetings, as well as in writing and through radio, television and the Internet. We know of no studies that have sought to evaluate the isolated effect of such psychoeducation on mental distress following a disaster. Psychoeducation is usually part of every mental health intervention following a natural disaster. Many of these interventions have proven effective in reducing the mental health sequelae of disasters. For a review of different programmes and their effectiveness, see Pfefferbaum and co-workers (2014a, 2014b). A study that provides pre-event general psychoeducation to children about the effects of experiencing a disaster (or other potentially traumatic event) in a community that later experiences a disaster would provide valuable information about such interventions in the future.

### 4.4. Communication through web sites and social media

Considering the extensive use of the Internet, Ryan et al. (2012) argues that this arena can function as a unique tool in preparing children for disasters, especially those children living in disadvantaged homes where parents are less educated, less involved and where there are fewer resources to achieve efficacy and preparedness. Children can access disaster information that is not provided at home through the Internet. Moreover, online tools are cost-effective and may reach many children (Ryan et al., 2012). Studies are beginning to investigate the content of web sites aimed at increasing children’s disaster preparedness (Ryan et al., 2012), but little is known about the effects of such sites on children. It should be remembered that using the Internet as a tool can become a problem following disasters where there is extensive damage to a society’s infrastructure.

Some research looked at how social media can be used in disaster communications. The above-mentioned ‘zombie apocalypse’ disaster campaign became popular across social media platforms. Despite its success, the reported behavioural intentions to engage in emergency-preparedness behaviours were relatively low across conditions both in terms of emergency-preparedness behaviours as well as information-seeking behaviours (Fraustino & Ma, 2015). While the campaign was successful in rising awareness by creating a buzz, it was unsuccessful when it came to changing people’s behaviour. It will be a challenge to make a campaign that both creates a buzz and prompts behavioural changes. Considering the existing research about dissemination of risk information and social and peer influence, research should investigate in more detail which norms affect and influence children’s and adolescents’ risk perceptions. Knowing more about how children and adolescents communicate with each other about disaster risk can help us to create better disaster preparedness and more effective interventions directed towards youth.

## 5. Concluding remarks

There is limited research in the field of risk communication concerning children and adolescents. There are indications, however, that programmes should be implemented in schools to inform children about potential risks, teaching them how to be prepared for and act during a disaster and providing them with psychoeducation regarding normal reactions (before and) when a disaster strikes. Parents should be guided on how to talk to their children about risks and what they can do to be prepared. To reach many young people, the use of the Internet, campaigns and social media might be useful, and youth should be engaged and involved in the communication strategies due to the influence adolescents have on their peers.

Houston et al. (2016) concluded that empirical research on the effects of disaster communication on children and their families is essentially non-existent. We echo that. Although materials for disaster preparedness and psychoeducation have been developed, tests of their effectiveness are largely absent. Therefore, there is limited evidence regarding the effectiveness of the different formats, messages and campaign strategies (Houston et al., 2016). Two questions need to be addressed: Does increased disaster knowledge in children and adolescents lead to actual behaviour change during disasters? Does this knowledge foster resilience and coping?
